# NMDA receptor antibody encephalitis presenting as Transient Epileptic Amnesia

**DOI:** 10.1016/j.jneuroim.2019.01.011

**Published:** 2019-02-15

**Authors:** Sharon A. Savage, Sarosh R. Irani, M. Isabel Leite, Adam Z. Zeman

**Affiliations:** a Cognitive Neurology Research Group, University of Exeter Medical School, Exeter, UK; b Oxford Autoimmune Neurology Group, Nuffield Department of Clinical Neurosciences, University of Oxford, Oxford, UK; c Discipline of Psychology, University of Exeter, Exeter, UK

**Keywords:** Autoimmune encephalitis, Epilepsy, Neuroimmunology, NMDA, Memory

## Abstract

Transient Epileptic Amnesia (TEA) is a subtype of temporal lobe epilepsy, typically presenting in a person's early 60s, and of unknown aetiology. Encephalitis caused by antibodies to NMDA receptors (NMDARE) has not previously been documented in TEA. We describe a 47-year-old male who satisfied criteria for TEA, but given his atypical symptoms, was also screened for autoimmune epilepsy. High levels of serum NMDAR antibodies were found, suggesting NMDARE. Immunosuppressive treatment gradually eliminated the NMDA receptor antibodies. Our case extends the clinical spectrum associated with neuronal cell-surface autoantibodies to include atypical cases of TEA.

## Highlights

• Transient epileptic amnesia (TEA) is a recently defined syndrome of late life epilepsy.
• Its main manifestation is recurrent amnesia, often on awakening.
• NMDA receptor encephalitis (NMDARE) has a widening phenotype.
• We describe a case of NMDARE presenting as TEA.
• TEA is sometime caused by an autoimmune process.

## 1 Introduction

Transient Epileptic Amnesia (TEA) is a subtype of temporal lobe epilepsy which typically presents in people aged in their early 60s. It is characterised by repeated, brief (median 30 min) episodes of mixed anterograde and retrograde amnesia, often on awakening (Zeman and Butler, 2010). One third of patients have exclusively amnestic attacks; the remainder display additional manifestations of epilepsy (often olfactory hallucinations (Savage et al., 2017)). Interictal memory disturbances also frequently arise, typically including accelerated long-term forgetting (ALF) - the loss of normally acquired information over hours to weeks; a patchy autobiographical amnesia; and impaired memory for familiar routes and landmarks. The amnestic episodes respond promptly to antiepileptic drugs (Butler et al., 2007). The syndrome is an important differential diagnosis of memory loss in mid- to late-life.

Encephalitis caused by antibodies to NMDA receptors (NMDARE) was first recognised in 2007 (Dalmau et al., 2007). It was initially described primarily as a paraneoplastic disorder affecting young women with ovarian teratomas, presenting dramatically with a ‘viral’ prodrome followed by psychosis, cognitive dysfunction and seizures, often progressing to a distinctive movement disorder with dysautonomia and coma. Since the original description, presentations have widened, including cases with isolated psychosis, epilepsy or movement disorder, predominant dysautonomia or hypoventilation, in men, women and children, and mostly without a tumour (Irani et al., 2010). Structural imaging is usually unremarkable but resting state connectivity has shown bilateral hippocampal network dysfunction (Finke et al., 2013), and indeed, the hippocampus has the highest density of NMDARs in the human brain (Heine et al., 2015). Disease pathogenesis involves antibody-mediated internalisation of NMDARs with resulting NMDAR-hypofunction. Current treatment involves the use of high-dose corticosteroids, plasma exchange and/or intravenous immunoglobulin as first-line agents, plus rituximab, cyclophosphamide, as ‘add-on’ second-line therapies in refractory cases. The roles of mycophenolate mofetil (MMF) and azathioprine are poorly studied.

## 2 Case report

PT, a 47-year-old male shop-worker, presented in October 2012 at age 44 with recurrent amnesia on awakening, characterised by disorientation and repetitive questioning which would last for minutes, in conjunction with 30 second ‘trance-like’ episodes during the day. As reported by his partner, these daytime episodes were sometimes accompanied by repetitive swallowing movements, followed by disorientation and repetitive questioning, and occurred in clusters of up to seven per day. PT himself was aware of some but not all of these episodes, with partial recollection of being unable to remember. He did report other memory changes, however, including an unexpectedly rapid loss of newly acquired memories over days, and an unusual difficulty in recalling both autobiographical events from his adulthood and familiar routes. Concurrently, PT developed olfactory hallucinations; uncharacteristic headaches with migrainous features; postural arm tremor, tingling legs, malaise and low mood. The olfactory hallucinations, which reminded him of candy floss, occurred independently of the amnestic episodes and could last for up to a day. He also reported some subjective blunting of his sense of smell. His medical history was unremarkable, aside from symptoms of depression during the previous year and a remote history of heavy alcohol use.

A timeline of PT's symptoms and investigations is provided in Table 1. Initial brain MRI showed subtly increased T2 signal in the right hippocampus, in the absence of any recent episodes. EEG was normal. Clinical examination revealed no focal neurological signs. Following an initial clinical diagnosis of TEA, PT was treated with lamotrigine (commencing at 25 mg and increasing incrementally up to 100 mg twice a day), resolving his trance-like and amnestic attacks, but not his other symptoms (namely headaches, tingling sensation in the limbs, and olfactory hallucinations).

**Table 1:** Clinical timeline of symptoms and investigations.

Timeline	Event
Oct 2012	Onset of amnestic episodes, trance-like episodes and physical symptoms
May-June 2013	Lamotrigine commenced; amnestic seizures ceased; EEG (normal); MRI (subtle changes)
July-August 2013	One further amnestic seizure; NMDA positive (1:500); CT (normal); neuropsychological assessment (i) conducted
Sept-Oct 2013	Lamotrigine dose increased; no further seizures; intravenous steroids introduced plus 6-month oral maintenance therapy
Feb-March 2014	Partial improvement: NMDA positive (1:100), but headaches, olfactory hallucinations, numb fingers return
Mar-April 2014	Plasma exchange initiated plus steroids and mycophenolate - > substantial improvements in symptoms; NMDA negative
Oct-Nov 2014	Physical symptoms heightened (headache, pain); NMDA positive(1:100); 2nd course plasma exchange plus intravenous steroids provided
Jan-April 2015	Physical symptoms improve; NMDA negative
June-Aug 2015	MRI (normal); NMDA negative; some headaches; neuropsychological assessment (ii) conducted
Oct 2015-Mar 2016	Reported improvement in memory; no reported physical symptoms; NMDA negative

Although PT satisfied criteria for a diagnosis of TEA, in view of his relatively young age for a diagnosis of TEA, varied neurological symptoms and subtle MRI changes, the possibility of an autoimmune epilepsy was raised. Screening for associated antibodies revealed high levels of serum NMDAR antibodies (strong binding persisted at a dilution of 1:500, live cell based assay and rodent brain immunohistochemistry). LGI1, CASPR2, GABA(B)R- and AMPAR-antibodies were negative. CT scan of thorax, abdomen and pelvis and testicular ultrasound showed no evidence of malignancy. CSF NMDAR-antibodies (now recommended to support this diagnosis) were not measured, but the combination of amnesia, mood disturbance, seizures, a movement disorder, imaging findings and high serum NMDAR antibodies made a diagnosis of NMDARE likely.

**Fig. 1:**
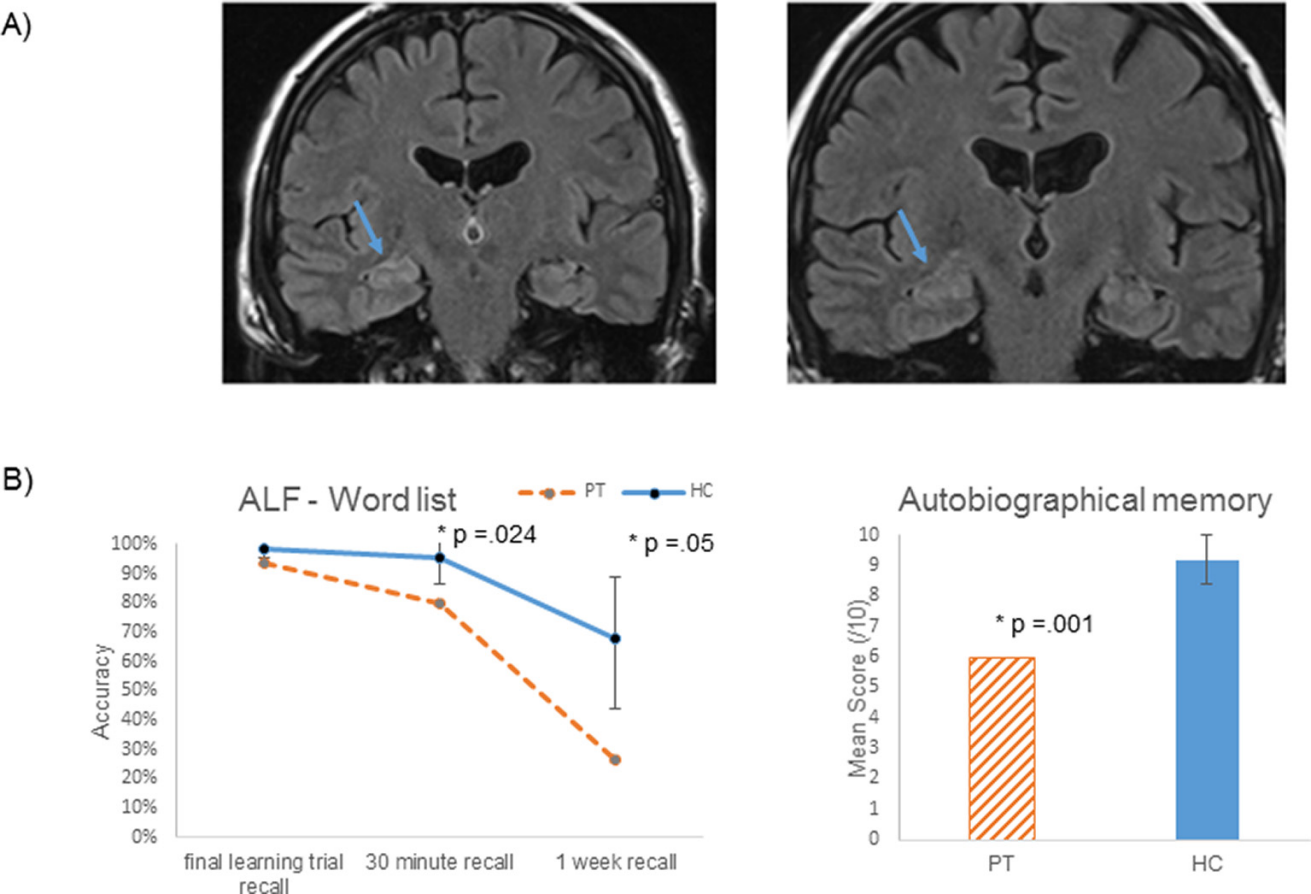
A) MRI showed subtle increase in signal in the right hippocampus in June 2013 which was no longer detectable in June 2015; B) Neuropsychological testing (2015) shows that PT can acquire new verbal information normally, but recall is significantly lower than *n* = 10 healthy controls (HC) after a 30-minute delay (Crawford's modified *t*-test: *p* = .024), with further reductions at one week (<30% retained) in comparison to healthy controls (where the average is more than double PT's score, *p* = .05). Likewise, PT's ability to recall rich, episodic details of events from different decades of his life is also substantially poorer than healthy controls (*p* = .001).

Initial treatment with intravenous methylprednisolone followed by oral maintenance therapy for 6 months led to partial resolution of symptoms, with a lower NMDAR-antibody level (1:100). Further treatment with plasma exchange followed by maintenance therapy with MMF and oral steroids substantially improved his symptoms and eliminated serum NMDA-receptor antibodies. Seven months later, relapsing symptoms were associated with the reappearance of the NMDAR-antibodies and a further course of intravenous methylprednisolone and plasma exchange was given. Repeat MRI now appeared normal (see Fig. 1a). PT is currently well on maintenance MMF, lamotrigine and a reducing dose of prednisolone.

Neuropsychological assessment was conducted in 2013 and 2015 using standard measures of: general cognitive ability (Wechsler Abbreviated Scale of Intelligence and Addenbrooke's Cognitive Examination-Revised – ACE-R), anterograde memory (Anna Thompson story from Wechsler Memory Scale 3rd edition, Rey Complex Figure Test), and executive function (verbal fluency tasks). Additional memory testing in 2015 included research measures of ALF (based on a modified administration of the Rey Auditory Verbal Learning test) and a modified Autobiographical Memory Interview (Zeman and Butler, 2010). Results for both 2013 and 2015 assessments indicated preserved general ability (estimated IQ = 109), with stable and preserved visuospatial skills, verbal and non-verbal reasoning, verbal generativity and retention of verbal material (stories) at 30-minute delay. Recall of visual information, however, was poor at both assessments. While initial encoding of information appeared to have worsened in 2015, PT now showed benefit from repeated exposure of material (e.g. in learning a name and address on the ACE-R). Further memory testing completed at the second assessment to evaluate accelerated long-term forgetting and autobiographical amnesia, indicated mild decrements in memory at standard delays, with evidence suggestive of accelerated forgetting after a 1-week delay, and pronounced autobiographical amnesia when comparing PT with 10 healthy older but IQ-matched controls (mean age = 59, IQ = 115) (Fig. 1b).

## 3 Discussion

The aetiology of TEA is unknown in most cases. There is no evidence for an elevated cerebrovascular disease burden, and the condition is not generally a precursor of dementia (Savage et al., 2016). While ALF and autobiographical amnesia have been described in paraneoplastic and autoimmune forms of limbic encephalitis (Witt et al., 2015), this is the first report which clearly documents TEA in the context of an autoimmune epilepsy. Our case further extends the widening clinical spectrum associated with neuronal cell-surface autoantibodies, indicating that autoimmunity should be considered not only when features specifically suggest an auto-immune aetiology, but also in cases where features are atypical for alternative diagnoses (such as relatively young age in TEA). It would be of interest in future work to assay CSF NMDAR-autoantibodies.

## Acknowledgments, competing interests, funding

This research was supported by The Dunhill Medical Trust [grant number R322/1113] and by the National Institute for Health Research (NIHR) Oxford Biomedical Research Centre (BRC). The views expressed are those of the author(s) and not necessarily those of the NHS, the NIHR or the Department of Health). SRI is a co-applicant and receives royalties on patent application WO/2010/046716 (U.K. patent no., PCT/GB2009/051441) entitled ‘Neurological Autoimmune Disorders’. The patent has been licensed to Euroimmun AG for the development of assays for LGI1 and other VGKC-complex antibodies with royalties paid to Euroimmun AG. SRI is supported by the Wellcome Trust (104079/Z/14/Z), BMA Research Grants- Vera Down grant (2013) and Margaret Temple (2017). The authors declare that they have no financial or other conflicts of interest in relation to this research and its publication. Informed consent was obtained from PT (not real initials), with study approval by the Multicentre Research Ethics Committee, United Kingdom (MREC 03/10/77).
